# Ligand binding site superposition and comparison based on Atomic Property Fields: identification of distant homologues, convergent evolution and PDB-wide clustering of binding sites

**DOI:** 10.1186/1471-2105-12-S1-S35

**Published:** 2011-02-15

**Authors:** Maxim Totrov

**Affiliations:** 1 Molsoft LLC,3366 N Torrey Pines Ct, La Jolla, CA 92037, USA

## Abstract

A new binding site comparison algorithm using optimal superposition of the continuous pharmacophoric property distributions is reported. The method demonstrates high sensitivity in discovering both, distantly homologous and convergent binding sites. Good quality of superposition is also observed on multiple examples. Using the new approach, a measure of site similarity is derived and applied to clustering of ligand binding pockets in PDB.

## Background

Experimental structural biology efforts are uncovering protein structures at unprecedented rate. There is a need to understand relationships and discover similarities between the solved structures. While fold comparisons are routinely performed to identify homologies that are at or beyond the limit of the sequence comparison methods, some functional relationships can only be detected at the level of binding sites. Ultimately, it is the configuration of these sites rather than overall sequence or fold, that determine enzymatic or signal transduction activity of a protein.

Most existing methods for binding site comparison are based on some form of coarse-grain representation of the geometry and properties of the pocket as a set of points or centers. Using a variety of algorithms, correspondence between the two sets is established. FLAP [1] algorithm first generates GRID [2] molecular interaction fields, which are used to detect locations where interactions of chemical groups with particular pharmacophoric features would be most favorable. Four-point pharmacophores are constructed from these points and used for target site matching. PocketMatch [3] is an algorithm for comparison of binding sites in a frame-invariant manner, based on representation of the sites by sorted lists of distances capturing shape and chemical nature of the site. Lists are compared using a special alignment algorithm and PMScore function. IsoCleft [4] detects 3D atomic similarities between binding sties using a graph-matching method. Protein functional surfaces [5] methodology attempts to optimize global shape and local physicochemical ‘texture’ match between a pair of surfaces using object recognition techniques. Often, search algorithm is combined with a specially compiled database of binding sites, for example CPASS database comprises ligand-defined binding sites found in the protein data bank (PDB) and CPASS algorithm compares these ligand defined sites to determine similarity without maintaining sequence connectivity[6]. Similarly, SURFACE is a database of protein surface regions, with finctional surface patches defined by sets of residues, and searches performed by matching the residue sets[7]. CavBase is a dataset of cavities extracted from PDB and searcheable using an algorithm that matches pseudocenters analogous to pharmacophoric points [8]. The Superimposé webserver [9] implements several superposition and comparison methods in an on-line format and allows detection of similarities between binding sites or entire proteins. A searchable database for comparing protein-ligand binding sites for the analysis of structure-function relationships has been reported [10], including comparison method based on geometric hashing, which identifies maximum common sub-graph of atomic features[11]. Med-SuMo rapidly compares protein surfaces represented by triplets of chemical groups[12]. Standard 3-, 4- and 5-point pharmacophores extracted from binding pockets identified by *icmPocketFinder*[13] across human PDB protein structures were used create a virtual library of sites in human pocketome, and querying the library with a pharmacophore of methyl-lysine binding site, interesting non-trivial hits were retrieved [14]. Of note, another perspective on the pocket comparison problem, which is to detect principal *differences* between related sites, was taken by several groups [15-17].

Discretized representation of the continuous pocket surface by amino-acid residues, chemical groups, pharmacophoric points or similar descriptors, allows very rapid comparison but may not be always adequate to capture distant similarities. Pharmacophoric points are well-suited to represent highly localized interaction centers, such as hydrogen bond donors and acceptors. Hydrophobic interactions and shape complementarity on the other hand are continuously distributed properties that lend themselves poorly to point representation. Moreover, to detect distant pocket similarities, ‘fuzzy’ matching may be needed because some of the discrete features may disappear, appear or change. These issues can be partially overcome by increasing the number of representative points and allowing partial matches.

**Figure 1:**
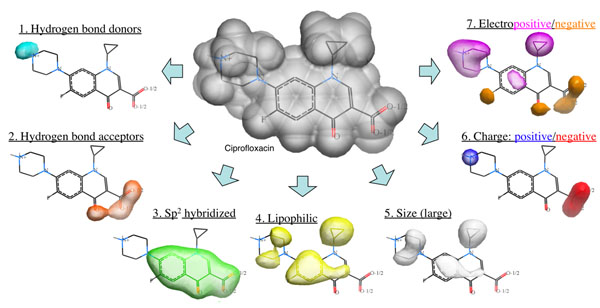
Seven components of the APF potential. Reproduced from ref. [18]

Ultimately, it might be a better solution to use continuous representation instead of discreet points. In the related field of ligand or small molecule superposition, a method using continuous pharmacophoric Atomic Property Fields (APF) has been recently proposed [18]. The method represents 7 atomic properties (hydrogen bond donor, hydrogen bond acceptor, lipophilicity, size, electronegativity, charge, aromaticity/hybridization) as continuous potentials projected in 3D space from atom centers using Gaussian functions (Fig 1). Each atom type is characterized by a distinct 7-component vector of properties, and a pseudo-energy reflecting similarity of 3D property distributions can be calculated for two or more molecules. By optimizing the APF pseudo-energy, optimal superpositions of multiple ligands that bring together identical or similar atoms and separating dissimilar ones can be identified. The approach was successfully tested in pairwise flexible ligand superposition and multiple chemical alignment. In a recent independent study, APF performance was compared to other small molecule superposition techniques and the method demonstrated best superposition accuracy across a large benchmark [19]. Promising results were also obtained in the application of APF potential to ligand-based virtual screening and 3D QSAR [18]. An optimized measure of chemical similarity based on APF was derived[20].

In the present work, the APF approach is adapted to the problem of binding site/pocket superposition. The resulting pocket superposition method is tested on multiple distantly similar pocket examples. The method also produces a score characterizing the degree of similarity of the pockets. The utility of the APF site superposition as a site comparison method is evaluated by calculating a complete distance matrix for the set of over 5000 binding sites in scPDB binding site database[21]. Finally, clustering of this available slice of the pocketome is performed.

## Methods

### Adaptation of the APF ligand superposition method to binding site superposition

The original APF ligand superposition protocol consists of (I) generation of grids with 7 APF potential components from the template (static) ligand and (II) optimization of the target ligand in the grid APF potentials combined with internal force-field energy of the ligand. Monte-Carlo with gradient minimization after each random step is used as a global energy optimizer. Six variables controlling overall position of the ligand as well as torsions around rotatable bonds are optimized.

**Figure 2:**
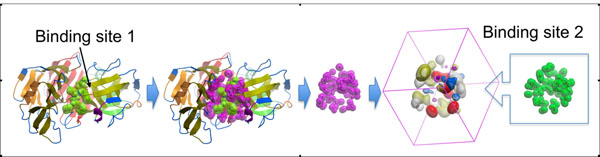
Diagram of APF BSS protocol.

Here, the binding site was defined as a collection of receptor atoms carved by a sphere around the ligand found in the X-ray structure (6Å radius was chosen based on the results of preliminary tests). One of the two sites to be superimposed was used to generate 7-component APF potentials on a grid (0.5Å spacing). The second site was placed in these pre-calculated grid potentials and the system was subjected to Monte-Carlo minimization procedure to find optimal superposition. The site was treated as rigid and therefore only six positional variables needed to be optimized, three polar coordinates that define position of the center of mass and three angles that define the orientation. Pseudo-Brownian Monte-Carlo sampling with local gradient minimization (100 steps) after each random step was used [22,23]. Effective temperature in Metropolis criterion was set to 5000K and simulation was terminated after 10,000 energy evaluations. The entire atomic property field binding site superposition (APF BSS) protocol is implemented as a script in ICM [24,25]. Schematic outline of the protocol is represented on Figure 2.

### Distance matrix calculation and clustering

APF pseudo-energy or score E_APF_ for the optimal superposition reflects the similarity of the atomic property distributions of the two binding pockets. It can be used directly for ranking of the database binding sites by their similarity to a query. However, for some other applications such as clustering, it is necessary to derive a similarity measure that behaves distance-like, rather then ranking score-like. In particular, for a pair of non-identical sites it has to be a positive value that increases as they become more dissimilar and becomes zero for identical pairs. On the other hand, E_APF_ is always negative, and the value for identical sites varies depending on the size and composition of the site. To convert E_APF_ to a normalized dot product-like measure with a correct asymptotic behavior, we used the following formula:

S_APF_ = *tanh*((E_APF_-E_0_)/∆_0_),

where E_0_ and ∆_0_ are empiric parameters. Next, distance-like similarity measure is obtained from dot-product-like:

D_APF_(A,B)=(S_APF_(A,A)+S_APF_(B,B))-2S_APF_(A,B)

**Figure 3:**
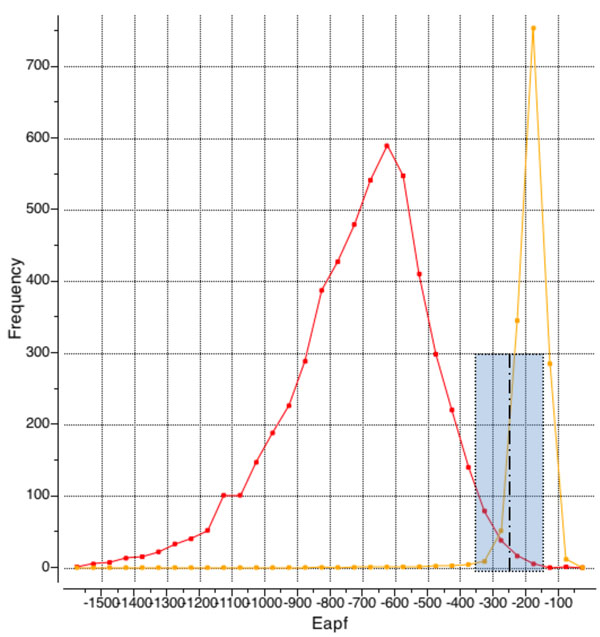
Histograms of optimal E_APF_ for random site pairs (orange) and identical pairs (red).

Estimates of E_0_ and ∆_0_ parameters were deduced from the statistics of the APF scores for identical and random site pairs (Fig. 3) Observed distributions suggested ∆^0^=100 and E^0^=-250. The resulting distance matrix was used as input for UPGMA (Unweighted Pair Group Method with Arithmetic Mean) clustering algorithm[26].

## Results and discussion

**Figure 4:**
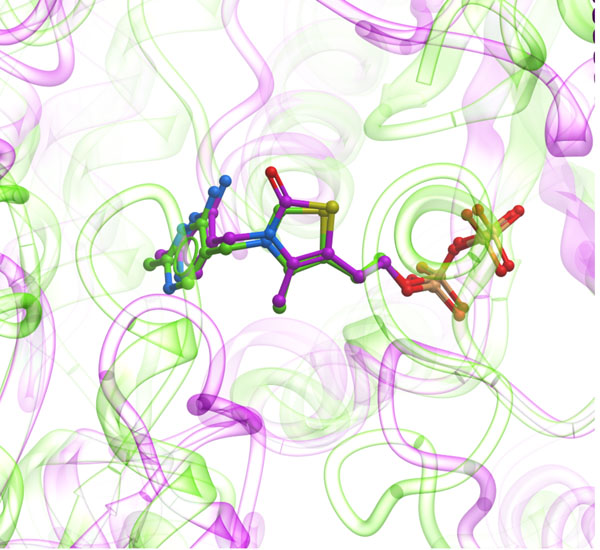
Example of the tight superposition of similar ligands upon APF superposition of their binding sites: thiamine diphosphate in the binding sites of pyruvate dehydrogenase (1rp7, magenta) and pyruvate decarboxylase (1pvd, green). The resulting RMSD for thiamine ligand in this example is only 0.52A. At the same time even superimposable segments of the receptors’ secondary structure (transparent ribbons) experience much larger displacements. Sequence identity between the two proteins is 19.2%

One indication of the accurate binding site superposition is that when the two pockets contain identical or similar ligands, they should become closely overlaid. Upon application of APF BSS to a variety of complexes, remarkably tight superposition of the ligands in the two structures was indeed observed, even though the ligands themselves didn’t play any explicit role in the BSS process (see an example on Fig. 4). We attribute this high accuracy to the emphasis the procedure places on superposition of the atoms and moieties that actually line the pocket rather than the underlying amino-acid residues and tertiary structure which may diverge dramatically.

**Figure 5:**
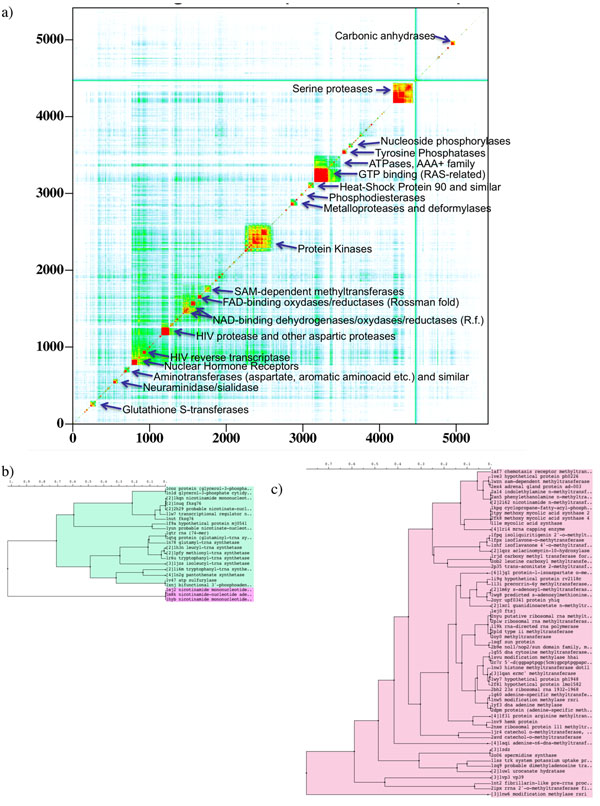
(a) Heat-map of the distance matrix between all binding sites in scPDB ordered accotding to the clustering tree. Warmer colors (red) correspond to closer APF similarity. Larger diagonal blocs are annotated. Because the complete tree is too large, to illustrate its structure the branches comprising SAM-dependent methyl transferases (b) and aminoacyl-tRNA synthases (c) are shown.

The ability of the APF BSS algorithm to detect and successfully superimpose distantly homologous binding sites was investigated by applying it to all pairs of sites within scPDB. Optimal APF superposition scores were used to generate a distance matrix and cluster the binding sites by similarity. To visualize the results, the distance matrix was plotted as a heat-map after re-ordering all sites according to the clustering tree (Fig 5). Major classes of enzymes formed easily identified clusters, with protein kinases by far the most represented family, followed by serine proteases and GTP-binding proteins.

Interestingly, a super-cluster emerged around GTP- and ATPases, grouping together other phosphatases, phosphorylases and phosphodiesterases, very likely due to common features associated with phosphate binding. Rossman fold-based NAD- and FAD- oxydases/reductases and SAM methyltransferases formed another large loose supercluster, having in common the adenine binding sub-site.

**Figure 6:**
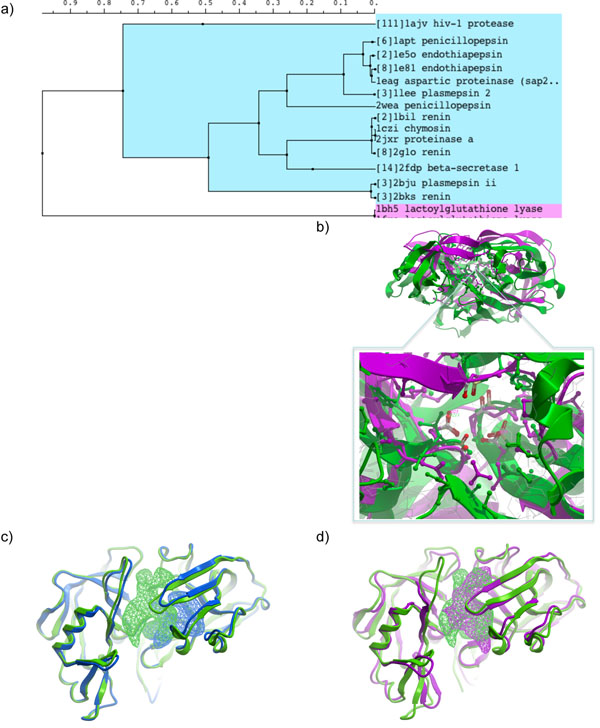
**(a)** APF clustering sub-tree containing aspartic proteases. Branches containing only multiple structures of the same protein are collapsed and the number of structures is indicated in square brackets. **(b)** Superposition of HIV protease and endothiapepsin. Closeup of the binding site reveals correct superposition of the catalytic aspartic acid pair. **(c,d)** Comparison of binding site pockets in two renin structures (1bil, green, and 2bks, blue), (c); and in chymosin (1czi, magenta) versus renin (1bil, green), (d). Due to alternative side-chain conformations and some backbone movement, very different binding pockets are seen in the two renin structures. The pockets in the chymosin/renin pair overlay much better, which explains why in the clustering tree 1bil and 1czi are adjacent while 2bks is on a relatively remote branch. Pocket blobs were generated using icmPocketFinder[13] and visualized in ICM.

It was instructive to review more in-depth a branch of the complete tree such as that containing various aspartic proteases. APF comparison and clustering correctly recognizes and puts together in this sub-tree multiple structures representing HIV protease, penicillopepsin, endothiapepsin, plasmepsin, renin, chymosin, proteinase A and beta-secretase binding sites (Fig 6a). Remarkably, correct 3D superposition of the active sites formed on the dimer interface (as in HIV protease) and within a single monomer (for example endothiapepsine) is produced (Fig. 6b), even though the overall sequence homology between the two proteins is negligible. Interestingly, multiple structures for the same protein do not always cluster directly together. Review of such cases revealed multiple binding modes and conformations that result in significant changes in the overall shape of the binding pocket. As a consequence, the binding pocket of a particular protein in certain structures may resemble stronger the pockets of other, homologous proteins rather than of the same protein in an alternative conformation (Fig 6c,d). Nevertheless, these diverse structures fall within the same aspartic proteases branch of the global site clustering tree, because despite the divergent shapes of the active sites they share key pharmacophoric features which are recognized by the superposition and comparison procedure.

**Figure 7:**
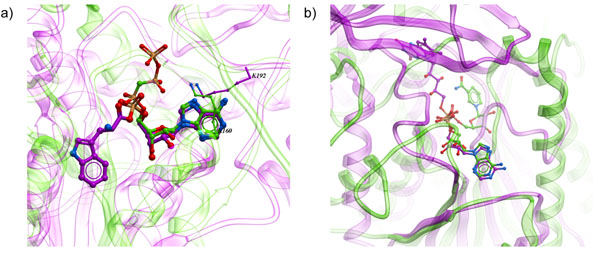
Example of APF superposition of the distantly homologous binding sites: **(a)** tryptophanyl-tRNA synthase (1i6m, magenta) and pantothenate synthase (1n2g, green). Despite divergent functions, substrates of both enzymes contain adenosyl moiety recognized by relatively conserved motifs. Also of note is the functional mimicry of certain side-chains belonging to different segments of the structure, such as K192 in 1i6m playing the role of K160 in 1n2g, both providing a hydrogen bond to the same nitrogen in adenyl moiety. Overall sequence identity of the two enzymes is 18%. **(b)** NAD binding site in UDP-galactose 4-epimerase (1ek5, green) and FAD binding site in D-amino acid oxydase (1ve9, magenta). The two enzymes share similar Rossman fold sub-domains binding adenosyl moiety, while their other sub-domains are very different. Parts of well-superimposed ?-?-?-?-? structure can be seen at the bottom of the figure (transparent ribbons).

Recurrent theme that could be observed in distantly related binding sites is the conservation of a sub-site recognizing common moiety in otherwise different substrates or co-factors. For example, in tryptophanyl-tRNA synthase and pantothenate synthase, similar sub-pocket binding adenosyl was detected. When the binding sites are superimposed by APF BSS procedure, the corresponding adenosyl portions of the ligands are overlayed near-perfectly (Fig 7a).

Similarly, FAD and NAD cofactors in in UDP-galactose 4-epimerase and D-amino acid oxydase share the same binding mode for the common nucleotide and this homology is successfully detected despite very different portions that coordinate flavine and nicotinamide (Fig 7b).

**Figure 8:**
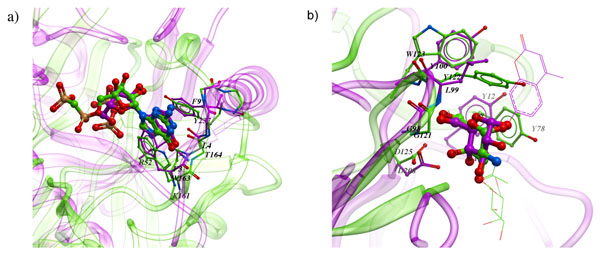
Examples of convergent binding sites on apparently unrelated enzymes, tightly superimposed by APF method: (a) GDP bound to gdp-mannose mannosyl hydrolase (1rya, magenta) and to calcium-dependent endoplasmic reticulum nucleoside diphosphatase (1s1d, green). Residues coordinating guanidyl moiety – the sidechains of K161, W163, Y237 and the backbone of T164 in 1rya align well in space and play the role of R52, F3, and F9 and the backbone of L4 in 1s1d. 1rya belongs to NUDIX hydrolase superfamily and alpha and beta fold class, while 1s1d is classified as apyrase and 5-bladed beta-propeller, according to CDD[27] and SCOP[28]. (b) Binding sites of concanavalin A (1cjp, magenta) and agglutinin (1jot, green). Despite lack of any overall homology, the two proteins bind the central sugar moieties (glucose in concanavalin A complex and galactose in agglutinin complex) of their ligands in a remarkably similar manner: beta-hairpins G98-L99-Y100 (concanavalin A) and G121-Y122-W123 (agglutinin) coordinate O5’ and O6 atoms via backbone hydrogen bonds; Y12 (concavalin A) and Y78 (agglutinin) engage aliphatic carbons on the opposite face of the sugar ring in hydrophobic interactions; D208 and D125 coordinate hydrogens on O4 and O6 hydroxyl oxygens. Parts of ligands other than the central sugar moiety are shown in wire representation for clarity.

Perhaps the most intriguing findings are the cases where similar binding mode is observed for the same ligand by two clearly unrelated receptors. APF BSS identified multiple such cases, two of which are illustrated on Figure 8. In both examples, not only similar side chains are lining the pockets, but also the backbone structure locally adopts similar conformation to form structurally convergent binding sites within otherwise unrelated protein folds.

## Conclusions

Sensitive and accurate binding site comparison is a technology with multiple important applications. Binding site databases could be screened for putative off-target sites for known or candidate drugs, either to discover and avoid side-effects or to find new applications. Functional annotation of ‘orphan’ pockets on newly resolved protein structures could be aided by identification of similar sites if known function. Initial drug design leads for new target proteins may be suggested by ligands binding similar sites in well-studied proteins. In contrast to previously reported methods, APF BSS utilizes continuous similarity measure and optimization algorithm which may identify and successfully superimpose distantly related sites missed by point-based approaches. Promising results in PDB-wide site comparisons illustrate sensitivity and accuracy of APF BSS.

## Competing interests

The author declares that he has no competing interests.
